# Predicting disease outcomes from remote monitoring using machine learning: a systematic review

**DOI:** 10.1186/s12911-026-03495-0

**Published:** 2026-04-25

**Authors:** Jonas Wolber, Andreas Schuppert, Martin Mücke, Julia Sellin

**Affiliations:** 1 Institute for Digitalization and General Medicine, Medical Faculty, RWTH Aachen University, Pauwelsstraße 30, 52074 Aachen, Germany; 2 Joint Research Center for Computational Biomedicine, Medical Faculty, RWTH Aachen University, Pauwelsstraße 30, 52074 Aachen, Germany; 3 Center for Rare Diseases Aachen, Medical Faculty, RWTH Aachen University, Pauwelsstraße 30, 52074 Aachen, Germany

**Keywords:** Chronic disease, Machine learning, Remote monitoring, Parkinson’s disease, Diabetes, Respiratory disease, Heart disease, Wearable devices, Explainable AI, Clinical decision support system

## Abstract

**Background:**

Chronic conditions cause millions of deaths annually worldwide. Remote patient monitoring using wearable devices and sensors, combined with machine learning (ML), offers promising strategies for disease management. However, diverse methodological approaches and study designs impede comparability and the development of best practice guidelines.

**Methods:**

A systematic review was conducted following the Preferred Reporting Items for Systematic reviews and Meta-Analyses (PRISMA) guidelines. Four scientific databases were searched for relevant prospective studies published between 2014 and 2024. Studies had to use ML to predict disease outcomes of chronic conditions in remotely monitored patients. The studies were tagged for characteristics such as health outcomes, dataset, monitored parameters, and algorithms.

**Results:**

From 6668 initially identified studies, 76 met inclusion criteria. 73.7% of studies were considered to have a high risk of bias, mainly due to methodological shortcomings in the Analysis domain. Parkinson’s disease was most frequently monitored, followed by diabetes and chronic obstructive pulmonary disease (COPD). Wearable devices were the predominant remote sensors, with accelerometer data being the most common parameter. Tree-based algorithms were most frequent, and studies using leave-one-out cross-validation showed significantly higher accuracy. Feature engineering and publication year were also significantly associated with model performance.

**Conclusion:**

This review highlights both progress and challenges in applying ML to chronic disease monitoring. While conditions like Parkinson’s, COPD, and diabetes are well-represented, others such as liver and kidney diseases are underexplored. Future research should prioritize standardization of methodologies, model interpretability, and ethical considerations including data privacy and algorithmic fairness. When properly implemented, ML-driven remote monitoring has the potential to enhance patient care, reduce complications, and deepen our understanding of chronic conditions. However, addressing challenges in reproducibility, generalizability, and clinical integration is crucial for advancing the field.

## Background

The burden of chronic conditions is a significant and growing concern in modern society. This includes cardiovascular disease, hypertension, respiratory diseases, type 2 diabetes, cancer, neurodegenerative disorders, and liver disease. These conditions are often negatively influenced by lifestyle choices such as poor diet, physical inactivity, smoking, excessive alcohol consumption, and chronic stress [1]. The impact of these conditions is immense, both on individuals and on healthcare systems worldwide. As an example, one of the most impactful chronic conditions is Diabetes. According to the International Diabetes Federation, as of 2021, an estimated 536.6 million people were living with diabetes globally, and this number is projected to rise to 783.2 million by 2045 [2]. According to the International Diabetes Federation, diabetes alone was responsible for an estimated 4.2 million deaths worldwide in 2019 [3]. Ischemic heart disease, COPD, dementias, and diabetes are among the top 10 leading causes of death worldwide [4]. These conditions are influenced, at least in part, by lifestyle choices, underscoring the significance of real-world data on lifestyle and disease progression.

Remote patient monitoring may help in the diagnosis and the detection of disease progression of chronic conditions as well as in monitoring lifestyle related factors due to its real life setting, and thereby facilitate better health outcomes. Remote monitoring devices such as smartwatches or mobile glucose sensors can monitor various vital parameters that can be used in the management of chronic conditions. For example, Wu et al. [5] used a combination of environmental parameters, wearable data and clinical questionnaires to detect acute exacerbations of COPD (AECOPD). Detecting AECOPD may significantly improve patient management by enabling early intervention, reducing hospitalization rates, and improving quality of life. Early detection allows healthcare providers to adjust treatment plans promptly, potentially preventing severe exacerbations and slowing disease progression. This proactive approach can lead to better long-term outcomes for COPD patients and reduce the overall burden on healthcare systems [6].

In addition to a growing number of applications in the medical field, wearable sensors are more and more adopted as a lifestyle choice by consumers [7], and wearable technology has improved as a result. This allows for more precise measurements as well as the measurement of ever more parameters such as ECG, blood oxygen saturation or energy expenditure in a real life setting [8]. The increase in measurable parameters as well as the increasing amount of users and consequently user data allows and also necessitates the use of machine learning (ML) for the interpretation of these wearable data. A promising approach is the prediction of disease outcomes from remote health monitoring data [9]. These studies have relied on different preprocessing methods, training strategies, and algorithms for their predictions. Both deep learning as well as more classical ML strategies have been successfully employed. However, while deep learning methods certainly have their advantages such as automatic feature extraction, they are also black-boxes making model interpretation often strenuous if not impossible [10]. A systematic review from 2023 [11] concluded that model interpretability as achieved by, e.g., white-box models, feature importance, or decision trees is essential for establishing ML in a clinical decision support system (CDSS) as it addresses safety concerns.

Most systematic reviews on remote monitoring to date have focused on specific diseases, such as heart failure [12] or COPD [13]. Castelyn et al. [14] conducted a review on prediction models for remote monitoring of chronic conditions (up to 2018), focusing on remote monitoring as an intervention. Shaik et al. [15] reviewed the general use of AI in remote monitoring, exploring monitoring methods, areas of application, and AI-related challenges. Peyroteo et al. [9] characterized 18 studies on remote patient monitoring in primary care. While these reviews largely adhered to the PRISMA reporting guidelines, a notable limitation was the absence of a structured risk of bias assessment tailored to prediction modeling. None of the studies applied the Prediction Model Risk of Bias Assessment Tool (PROBAST), which is specifically designed to evaluate the methodological quality and applicability of prediction models. In the context of machine learning (ML) studies, where results are highly sensitive to data characteristics, validation methods, and study design, applying PROBAST alongside PRISMA can enhance the interpretability and robustness of findings. Our review addresses this gap by combining both frameworks, offering a more comprehensive and reliable evaluation of study quality and model validity.

## Methods

### Objective

This systematic review aims to provide a comprehensive analysis of studies applying ML to remote patient monitoring for chronic diseases published between 2014 and 2024. Thereby, we show trends in applied ML algorithms, monitored conditions and outcomes, employed monitoring devices, data processing methods and more. By combining PROBAST risk of bias assessment with a comprehensive and detailed study characterization, we aim to elucidate current trends in this field, highlight research gaps, and uncover potential problems and opportunities in the field of remote chronic disease management and research through ML applications.

### Search strategy

We performed a systematic review in compliance with the guidelines for the Preferred Reporting Items for Systematic Reviews and Meta-Analysis (PRISMA). The Nested Knowledge Platform (http://www.nested-knowledge.com) was used for managing the articles and support with screening and tagging. Four research databases were searched for studies that use ML to predict an outcome of a chronic condition based on remotely gathered data: Pubmed, Scopus, Web of Science and Embase. Studies had to be peer-reviewed and published after January 1^st^ 2014. The eventual search string was a result of various preceding exploratory search strings and discussions between the authors to define and refine eligibility criteria and the scope of the review. The final search was performed on December 9^th^ 2024 and was applied to titles and abstracts. It was made up of three word groups relating to ML, remote monitoring and chronic conditions (see Table 1 for all search strings for according databases). We also manually added eligible studies by searching through references of included studies or related reviews.

**Table 1:** Search strings for the systematic review

Database	Search String
Scopus	TITLE-ABS-KEY (machine AND learning OR predict OR detect OR diagnose) AND (wearable OR home AND monitoring OR remote AND monitoring OR telemonitoring OR outpatient AND monitoring) AND (heart AND disease OR heart AND failure OR stroke OR hypertension OR copd OR asthma OR dementia OR parkinson OR cancer OR diabetes OR hyperglycemia OR hypoglycemia OR kidney AND disease OR liver AND disease OR cirrhosis OR arthritis) AND PUBYEAR$>$2013 AND PUBYEAR$<$2025 AND (LIMIT-TO (SRCTYPE, “j”)) AND (LIMIT-TO (PUBSTAGE, “final”)) AND (LIMIT-TO (DOCTYPE, “ar”))
Web of Science	AB$=$((machine learning or predict or detect or diagnose)) AND AB=(wearable or home monitoring or remote monitoring or telemonitoring or outpatient monitoring)) AND AB$=$(heart disease or heart failure or stroke or hypertension or copd or asthma or dementia or parkinson or cancer or diabetes or hypoglycemia or hyperglycemia or kidney disease or liver disease or cirrhosis or arthritis))
PubMed	(((“machine learning”[Title/Abstract] OR “predict”[Title/Abstract] OR “detect”[Title/Abstract] OR “diagnose”[Title/Abstract]) AND (“wearable”[Title/Abstract] OR “home monitoring”[Title/Abstract] OR “remote monitoring”[Title/Abstract] OR “telemonitoring”[Title/Abstract] OR “outpatient monitoring”[Title/Abstract]) AND (“heart disease”[Title/Abstract] OR “heart failure”[Title/Abstract] OR “atrial fibrillation”[Title/Abstract] OR “arrhythmia”[Title/Abstract] OR “stroke”[Title/Abstract] OR “hypertension”[Title/Abstract] OR “copd”[Title/Abstract] OR “asthma”[Title/Abstract] OR “dementia”[Title/Abstract] OR “parkinson”[Title/Abstract] OR “cancer”[Title/Abstract] OR “diabetes”[Title/Abstract] OR “hypoglycemia”[Title/Abstract] OR “hyperglycemia”[Title/Abstract] OR “kidney disease”[Title/Abstract] OR “liver disease”[Title/Abstract] OR “cirrhosis”[Title/Abstract] OR “arthritis”[Title/Abstract]) AND 2013/01/01:2024/12/31[Date - Publication]) NOT (“review”[Publication Type] OR “systematic review”[Filter])) AND ((humans[Filter]) AND (2014:2024[pdat])) AND (humans[Filter])
Embase	(‘machine learning’:ab OR predict:ab OR detect:ab OR diagnose:ab OR ‘risk assessment’:ab) AND (wearable:ab OR ‘home monitoring’:ab OR ‘remote monitoring’:ab OR telemonitoring:ab OR ‘outpatient monitoring’:ab) AND (‘heart disease’:ab OR ‘heart failure’:ab OR stroke:ab OR ‘atrial fibrillation’:ab OR arrhythmia:ab OR hypertension:ab OR copd:ab OR asthma:ab OR dementia:ab OR parkinson:ab OR cancer:ab OR diabetes:ab OR ‘kidney disease’:ab OR ‘liver disease’:ab OR cirrhosis:ab OR arthritis:ab) AND ‘article’/it AND (2014:py OR 2015:py OR 2016:py OR 2017:py OR 2018:py OR 2019:py OR 2020:py OR 2021:py OR 2022:py OR 2023:py)

### Study selection

The articles found from the literature search were independently screened and tagged by two researchers (J.W., J.S.). Screening occurred in a two-step process whereby first the title and abstract and second the full text was screened for eligibility criteria. In case of disagreement in the screening or the tagging process the two researchers discussed the issue and came to a common decision.

Studies were included if they used ML to predict outcomes for chronic conditions in a prospective, real-world setting, with most patient monitoring done outside of hospitals or labs. Studies using remotely gathered data, even if combined with lab data, were eligible. Simulated environments and synthetic data were excluded. Studies using additional not remotely gathered data were allowed. Eligible algorithms included classical ML algorithms, deep learning, ensemble models, and Bayesian approaches. Studies had to describe data origin, ML methodology, and report metrics. Proprietary algorithms without details were excluded. The review focused on ten chronic conditions: heart disease, hypertension, COPD, asthma, cancer, dementia, Parkinson’s, diabetes, kidney, and liver disease. Congenital diseases and post-surgery monitoring were excluded. Relevant outcomes included disease detection, severity, risk, exacerbations, readmission, mortality, medication status, and wellbeing. Irrelevant outcomes were events like coughing, eating, or glucose/heart rate forecasting. Studies were excluded if the study was carried out retrospectively, monitoring was an intervention, used synthetic data, were not written in English or lacked full-text availability. All inclusion criteria are summarized in Table 2.

**Table 2:** Inclusion criteria for studies in this systematic review

Inclusion Criteria
Patients monitored in a remote setting
Study focuses on disease outcomes related to the chronic conditions heart disease, hypertension, COPD,asthma, cancer, dementia, Parkinson’s, diabetes,kidney, and liver disease.
Relevant disease outcome monitored
Peer-reviewed article published between 2014 and 2024
ML applied and ML methodology sufficiently described
Prospective study design
Not related to surgery-related outcomes or congenital conditions
Includes data analysis and results
Monitoring is not used as an intervention
Study is in English
Full-text available

### Risk of bias assessment

PROBAST [16] is specifically designed for systematic review evaluating studies developing or validating prediction models and consists of four key domains: participants, predictors, outcome, and analysis. Each domain in PROBAST was rated as having “low,” “high,” or “unclear” risk of bias based on the PROBAST signaling questions. A study was classified as having a low overall risk if all domains were rated low, unclear if any domain was unclear and no domain was rated with high RoB, and high if one or more domains were rated high. Two independent reviewers (J.W., J.S.) conducted the risk of bias assessment, and disagreements were resolved through discussion.

### Data extraction

Included studies were characterized based on five aspects:

#### General study characteristics

Studies were tagged for the country of the first author’s institute and year of publication.

#### Health outcomes

Studies were tagged for condition, health outcome (disease exacerbation, diagnosis, severity, risk, hospitalization, mortality, patient wellbeing) and outcome labeling method (patient reported outcomes, medical diagnosis, event outcome, clinical test result, parallel measurement, forecasting, expert labeling).

#### Dataset characteristics

Monitoring duration (one day to four months), frequency (hourly to less than daily), and dataset size were reported. For studies with dropout, only patients used for model training and evaluation were considered for the dataset size.

#### Remote data sources and parameters

Studies were categorized by employed remote data sources (wearable, active measurement device, questionnaire, environmental sensor) and monitored parameters used for prediction.

#### Algorithms

Prediction algorithms were tagged by model type. When multiple models were mentioned, the best-performing model was selected. Validation strategies (k-fold crossvalidation, Leave-one-out crossvalidation (LOOCV), external, other, simple), data processing methods, evaluation metrics, and availability of code and data were tagged. For data processing methods we tagged studies using manual feature engineering (statistical or domain-driven feature creation prior to modeling), signal denoising, feature selection / dimensionality reduction, class downsampling, missing data imputation, outlier detection and data augmentation. We did not annotate other data preprocessing techniques such as data normalization or standardization.

For some study characteristics, multiple tags could be applied, e.g. multiple tags were applied for the category parameters when a study monitored multiple parameters such as heart rate, step count and body temperature simultaneously. Data extraction was performed by one reviewer (J.W.).

### Statistics

To investigate associations between study characteristics and model prediction performance, we conducted independent samples t-test for categories with only two groups and Analysis of variance (ANOVA) test for characteristics with more than two groups. We categorized the continuous variables publication year (2014–2017, 2018–2021, 2022–2024) and dataset size ($<$10, 10–50, 50–100, $>$100 samples). The four most common data processing methods (feature engineering, signal denoising, downsampling, and feature selection) were binarized (0 for non-use, 1 for use). We focused on studies reporting classification tasks and at least one of the four reported evaluation metrics: AUC score, accuracy, sensitivity, and specificity. For all statistical tests, we set the significance level to 0.05.

## Results

### Study selection

**Fig. 1:**
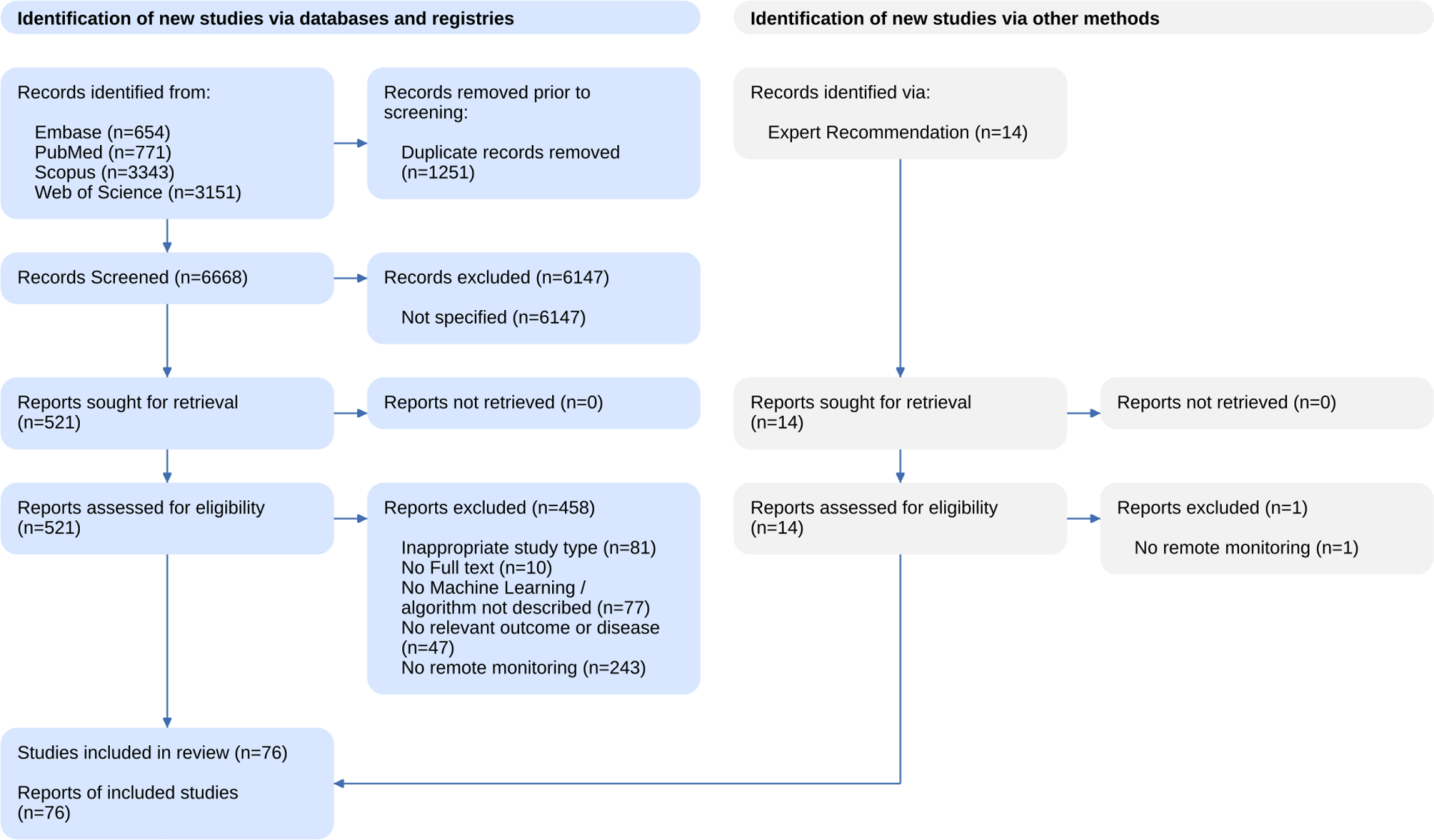
Flow diagram of the study selection process

Applying the search string, 6668 unique studies were found. From these we excluded 6147 studies after screening for title and abstract. 521 studies were further considered for full-text screening according to the eligibility criteria. After full-text screening, 62 studies met the eligibility criteria. 14 studies were added manually after searching for additional studies. Thus, in total, 76 studies were included in this systematic review (see Fig. 1 for an overview of the study selection process).

### General study characteristics

All included studies are listed in Table 3. Regarding the year of publication of the selected studies, 45 of the 76 studies were published after 2019, with 2024 (up to December 9^th^) being the year with the most included publications (13 studies).

**Table 3:** Study characteristics of selected studies

Author	Year	Out-comes	Data-set Size	Model
**Asthma**				
Huffaker [17]	2018	DE	16	RF
Lugogo [18]	2022	DE	298	GB
Emeryk [19]	2023	DE	149	RF
Alharbi [20]	2023	DE	10	GB
**Cancer**				
Liu [21]	2023	DM	40	GB
**COPD**				
Wu [5]	2021	DE	67	RF
Fernández-Granero (1) [22]	2014	DE	16	PNN
Fernandez-Granero (2) [23]	2015	DE	16	SVM
Burton [24]	2014	DE	19	LG
Tiwari [25]	2021	DE, DS	35	IF
Mohktar [26]	2015	DE	21	CART
Sanchez-Morillo [27]	2015	DE	15	KM
Shah [28]	2017	DE	110	LG
Jacobson [29]	2023	DE	18	RF
Merone [30]	2017	DE	22	PN
van der Heijden [31]	2014	DE	10	BN
Kolozali [32]	2023	DE	106	PLCA
Bhalla [33]	2023	DE	8	CNN
Yin [34]	2024	DE	66	GB
**Dementia**				
Lim [35]	2022	DD	18	DNN
**Diabetes**				
Dave [36]	2020	DE	112	RF
Zhu [37]	2022	DE	12	RNN
Georga [38]	2016	DE	15	KAF
Cvetković [39]	2016	DE	52	LG
Marling [40]	2016	DE	1	SVM
Maritsch [41]	2020	DE	1	GB
Bertachi [42]	2020	DE	10	SVM
Jahromi [43]	2023	DE	33	EM
Gu [44]	2017	DE	112	RNN
Elhadd [45]	2020	DE	13	GB
Deng [46]	2021	DE	40	CNN
Lehmann [47]	2023	DE	22	DT
Aljihmani [48]	2022	DE	32	EM
Montaser [49]	2024	DR	42	SVM
Khalilnejad [50]	2024	DR	200,000	GB
**Heart disease**				
Tison [51]	2018	DD	183	DNN
Mlakar [52]	2018	WB	24	RF
Aydemir [53]	2020	DS	43	SVM
Zhang [54]	2015	DD	34	SVM
Cuba Gyllensten [55]	2016	DE	91	MACD
Meng [56]	2020	WB	182	RF
Marzec [57]	2018	DD	235	RF
Larburu [58]	2018	DE	242	NB
Stehlik [59]	2020	DH	100	SBM
Wasserlauf [60]	2019	DD	24	CNN
Zhu [61]	2022	DD	204	HM
Cha [62]	2024	DD	13516	CNN
Hinrichs [63]	2024	DH	1538	DNN
**Hypertension**				
Ni, H. [64]	2018	DD	48	SVM
**Parkinson’s disease**				
Safarpour [65]	2022	DS	31	LM
Fisher [66]	2016	DE	34	DNN
Raknim [67]	2016	DD	17	SVM
Arroyo-Gallego [68]	2018	DD	52	EM
Arasteh [69]	2023	MS	12	CCA
Knudson [70]	2020	DS	34	LR
Rastegari [71]	2022	DD	60	SVM
Matarazzo [72]	2019	DS	59	RNN
Zhan [73]	2018	DS	129	RB
Evers [74]	2020	MS, DD	42	EN
San-Segundo [75]	2020	DE	6	CNN
Arora [76]	2015	DD, DS	20	RF
Molparia [77]	2018	DD	67	LR
Heijmans [78]	2019	DE	20	LG
Hammerla [79]	2015	DE	34	DNN
Zhang [80]	2020	DE	6	SVM
Zampogna [81]	2024	DS	71	LG
Hinchliffe [82]	2024	DE	72	SVM
Olaru [83]	2024	DE	16	LG
Shane [84]	2024	DD	132	RF
Crowe [85]	2024	DE	24	CNN
Andelman-Gut [86]	2024	DD, DS	24	SD
**Rheumatoid arthritis**				
Rao [87]	2023	WB	278	HMM
Gossec [88]	2019	DE	155	BN
Creagh [89]	2024	DS, DD	58	LG
Hinchliffe [82]	2024	DE	72	SVM
Looijen [90]	2024	DS	1208	LG

Abbreviations: CART = classification and regression tree, CCA = canonical-correlation analysis, CNN = convolutional neural network, DD = disease detection, DE = disease exacerbations, DH = disease hospitalisation, DM = disease mortality, DNN = deep neural network, DS = disease severity, DT = decision tree, EM = ensemble model, GB = gradient boosting tree, HM = hybrid model, HMM = hidden markov model, IF = isolation forest, KAF = kernel adaptive filters, KKN = K-nearest neighbor, KM = K-means clustering, LG = logistic / linear regression, MACD = moving average convergence/divergence, NB = naive bayes, PN = petri net, PLCA = probabilistic latent component analysis model, PNN = probabilistic neural network, RB = rank-based ML, RF = random forest model, RNN = recurrent neural network, SBM = similarity-based modeling, SD = subspace discriminant classifier, SVM = support vector machine, WB = patient wellbeing

Studies were mainly published either in Europe or the USA with the USA being the country with the most included publications (25 studies), followed by the UK (10 studies) and Spain (8 studies). China and Taiwan (3 studies each) were the countries with the most studies outside of Western countries.

### PROBAST risk of bias assessment

**Fig. 2:**
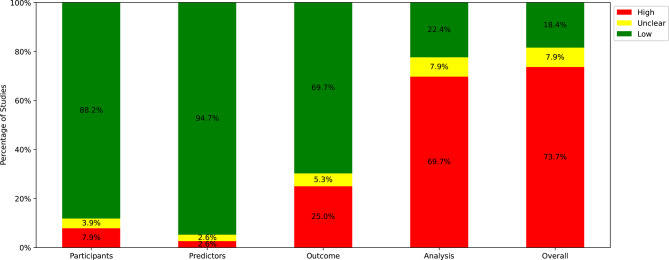
Distribution of risk of bias per domain and overall

Of the 76 studies analyzed, 55 studies were diagnostic in nature, while 20 studies were prognostic. In terms of study design, most studies (67 studies) focused solely on model development, while 8 studies included both development and validation phases, and only one study was dedicated to validation alone [59]. The overall risk of bias assessment revealed concerning results, with 56 studies (73.7%) classified as high risk, 14 studies (18.7%) as low risk, and six studies (8.0%) having unclear risk. Among the four PROBAST domains, the analysis domain emerged as the most problematic, with 53 studies (69.7%) showing high risk of bias. Another considerable source of bias was the outcome domain where 19 studies (25.0%) were at a high risk of bias. Figure 2 provides a graphical representation of these findings.

Detailed examination of the signaling questions revealed that of the 10 questions answered with ‘No’ or ‘Probably no’ (indicating high risk of bias), five belonged to the analysis domain. Notably, signaling question 4.1 (“Were there a reasonable number of participants with the outcome?”), was the most frequently identified source of bias across all studies (43 studies with ‘No’ or ‘Probably no’). For a comprehensive overview of the risk of bias assessment for each individual study and signaling question, readers are referred to Figure S1 in the Supplementary data.

### Health outcomes

In total, nine of the eleven selected conditions included in our search string were monitored in the selected studies. We did not find any studies monitoring patients with chronic kidney or chronic liver disease. Parkinson’s disease (PD) was the most frequently monitored and analysed condition with 22 studies, followed by Diabetes with 15, COPD with 14 and Heart disease with 11 studies. Rheumatoid arthritis (6), asthma (4) [17–20], dementia (1) [35], cancer (1) [21] and hypertension (1) [64] were less frequently studied. For Diabetes and heart disease we also investigated subconditions. From the 14 studies investigating Diabetes patients, 9 studies focused on Diabetes Type 1, two studies on Diabetes Type 2 [45, 46] and four studies did not select for any subgroup of Diabetes patients [39, 44, 47, 50]. In the eleven studies monitoring heart disease patients, seven studies investigated heart failure, three atrial fibrillation [51, 60, 61], one ventricular tachycardia [57] and one did not further specify [62].

Regarding the health outcome type, 40 of the 76 studies predicted disease exacerbations. Disease detection models were applied in 16 studies, followed by disease severity estimation in 11, patient well-being [52, 56, 82, 87] and patient medication status [69, 72, 74, 79] in four each, disease hospitalisation in two [59, 63], and disease mortality in one study [21]. The most often considered condition-outcome combinations were Diabetes exacerbations and COPD exacerbations with 13 each.

For labeling of health outcomes, most studies used patient reported outcomes (30 studies) to determine the true condition (the label for the ML model). Medical diagnosis was used in 12, event outcomes in 11, clinical test results and parallel measurement in 10, forecasting in 6 and expert labeling in 4 studies [19, 30, 61, 74].

### Dataset characteristics

There was a large variety in the dataset size of the studies. While three studies used only one diabetic subject to predict hypoglycemic events [40, 41, 67], another study used data from more than 200,000 patients to identify patients at risk of developing Diabetes [50]. The median dataset size of all studies was 37 while the heavily skewed average was at 2904. 19 studies had a dataset size greater than 100.

Another study characteristic we investigated was the average patient monitoring duration. 45 studies monitored their patients for longer than a month, 12 studies between one and four weeks, nine studies between one and seven days and four studies for only one day [64, 69, 74, 85]. For five studies [20, 46, 51, 53, 68] the average monitoring duration could not be determined.

Besides the monitoring duration, we also assessed the monitoring frequency. 54 studies monitored their patients more or less continuously with wearable devices, sensing accelerometer data or vital parameters in frequencies ranging from milliseconds to minutes. 15 studies obtained patient information on a daily basis, typically via questionnaires. In seven studies, patients were monitored less than once a day. For example, Jacobson et al. [29] collected digital health diaries and required only 76 entries within one year for a patient to meet inclusion criteria for their ML model.

### Remote data sources and parameters

53 studies employed a wearable device to monitor patients. Such monitoring devices included smartwatches, implantable cardioverter defibrillators and glucose monitoring devices. 20 studies used active measurement devices such as pulse oximeters, weight scales or blood pressure measurement devices. Additionally, 20 studies used patient questionnaires for collection remote patient data. Finally, five studies used environmental sensors such as a bed sensor or a home air quality sensing device [5, 17, 20, 32, 52].

**Fig. 3:**
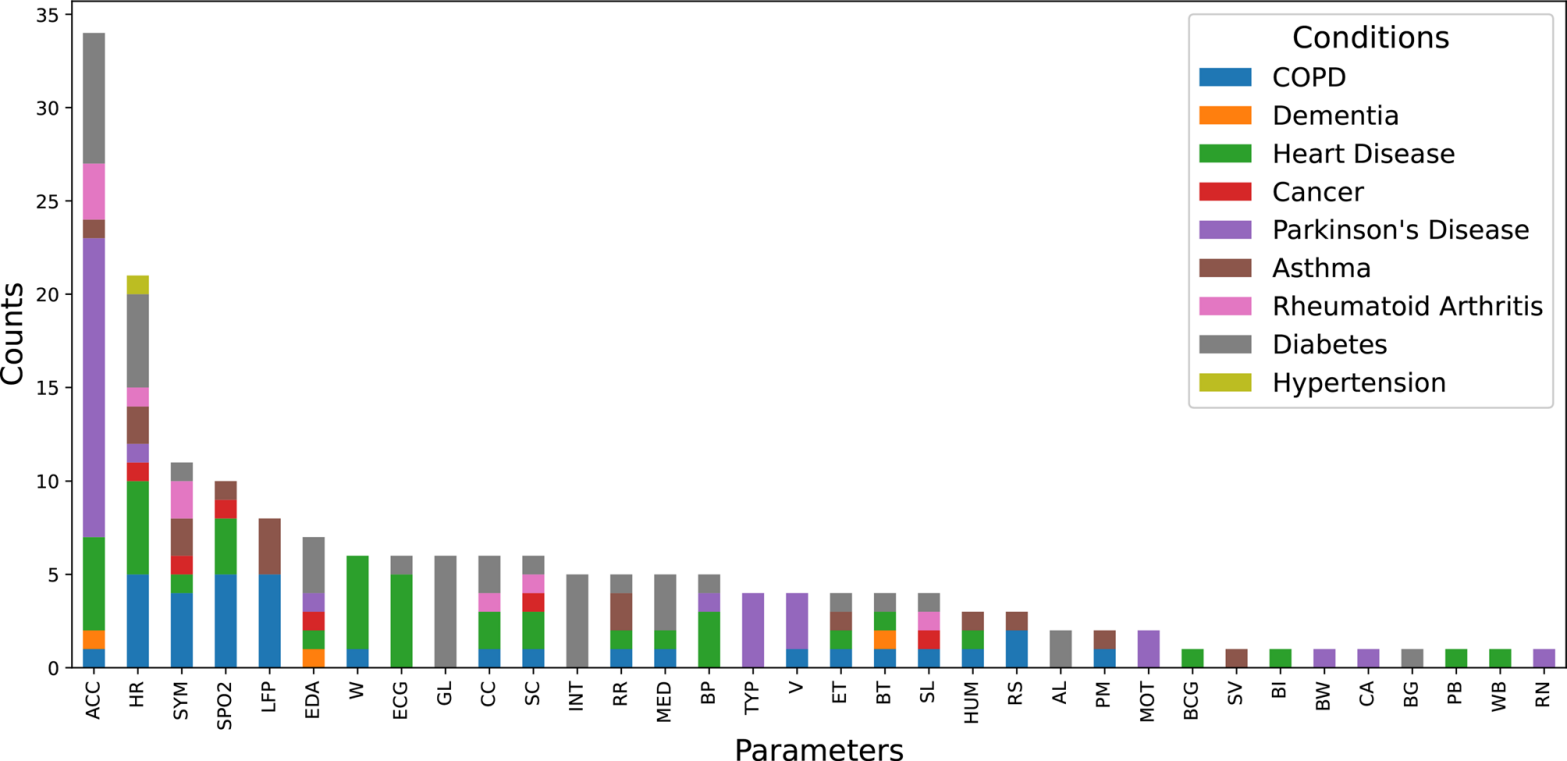
Parameters used for monitoring of chronic conditions. Abbreviations. ACC: accelerometer data, AL: activity logging, BCG: Ballistocardiogram, BI: bioimpedance, BP: blood pressure, BT: body temperature, BW: brain waves, CA: clinical assessment, CC: calorie consumption, ECG: electrocardiogram data, EDA: electrodermal activity, ET: environmental temperature, GL: glucose, HR: heart rate, HUM: humidity, INT: meal and drink intake, MED: medication intake data, MOT: motor function, PEF: peak expiratory flow, PM: fine particulate matter / air quality, RN: respiratory nasal airflow, RR: respiratory rate, RS: respiratory sound, SC: step count / distance walked, SL: sleep data, SPO2: oxygen saturation, SV: stroke volume, SYM: symptom data, TYP: finger tapping characteristics, V: voice, W: body weight, WB: patient wellbeing

The most frequently measured independent parameter was raw accelerometer data measured in 33 studies, followed by heart rate in 21 and symptoms in 11 studies. Overall, we annotated 34 different parameters. Note that for simplicity we generalized some parameters (e.g. lung function related parameters such as forced expiratory volume or peak expiratory flow were represented by the parameter lung function parameter). We further investigated how parameters related to the monitored conditions. Although accelerometer data were used in the most studies it was only used in seven different conditions, whereas heart rate was used in eight different conditions. Vice versa, the condition with the most different monitored parameters was heart disease with 19 parameters. In contrast, only 9 different parameters were used to monitor PD. See Figure 3 for an overview of parameters and the conditions monitored by them.

### Algorithms

We further investigated the prediction algorithms used in the studies. Out of all studies, tree-based models such as random forest or gradient boosting algorithms were most often used (19 studies), with random forest models being the most prevalent (10 studies). Twelve studies applied deep neural networks for their predictions. Of these, six studies used convolutional neural networks (CNN) and two studies used recurrent neural networks (RNN) [44, 61]. Less frequently used ML models included logistic / linear models (13), support vector machine models (11) and Ensemble models (3). These ensemble models were composed of random forest, support vector machine and k-nearest neighbor models [43], bagged tree ensemble [48] and an ensemble linear support vector regressor model [68]. 38 studies included interpretations of models by means of SHAP values, feature importance or displaying coefficients of models. The majority of studies utilizing tree-based models provided model interpretations (16 out of 19). For example, by interpreting their random forest model, Meng et al. [56] found out that step count / walking distance had the highest importance for classifying the self-reported health status of heart disease patients. In contrast, many studies using deep neural networks (5 out of 12), linear/logistic models (5 out of 13) and SVMs (5 out of 11) were not interpreted.

64 out of 76 of studies used some sort of crossvalidation method and three studies used external validation [51, 61, 63] to assess the performance and robustness of their prediction models. In 35 studies, a form of k-fold crossvalidation (e.g., 10-fold crossvalidation) was implemented. 15 studies used LOOCV.

Most studies used some form of feature engineering (54 studies). For continuous time series, mathematical transformations or calculations were used to derive features. For example, one study, investigating COPD exacerbations via respiratory sounds, applied a short time frequency transform filter [23]. In contrast, Gu et al. [44] used mathematical calculations based on physiological and temporal models of glucose and insulin dynamics to predict hyper- and hypoglycemia in diabetes patients. Signal denoising or smoothing (20 studies) was another common processing step, particularly for time series data. For example, Jahromi et al. [43] used a Butterworth filter to smooth out raw accelerometer data for detection of hypoglycemia from hand tremors. While we did not specifically tag whether datasets were imbalanced, we noted that 7 studies explicitly applied class downsampling to address class imbalance. Additionally, 4 studies [33, 46, 82, 85] used data augmentation. Other data processing steps included feature selection / dimensionality reduction (14), missing data imputation (9) and outlier detection (5) [20, 24, 26, 53, 83].

We further investigated whether studies shared their code or data. Eight studies openly shared the code of their implemented algorithm or shared anonymized patient data. 18 studies mentioned that data or code could be sent upon request. The majority (50 studies) did not share the code or the data in the publication.

Lastly, we also investigated the metrics for evaluation of model performance used by the different studies. AUC score was the most often displayed metric (39 studies), followed by specificity (38 studies), sensitivity (37 studies) and accuracy (31 studies).

### Association of study characteristics with performance

Although one has to keep in mind that performance validation of ML models can be heavily confounded by factors such as prediction horizon or dataset size, we next investigated the association of certain study characteristics with model prediction performance without accounting for any confounding factors. Table 4 shows the group means as well as *p*-values of the ANOVA test or the t-test for the selected study characteristics. We found that studies that performed no feature engineering reported signficantly higher values for sensitivity (*p* = 0.031) and accuracy (p$<$0.001). We provide an analysis on potential confounders between feature engineering and accuracy in the supplementary data (see section 6.2). In contrast, performing downsampling was associated with a significantly higher AUC score (*p* = 0.010) and performing feature selection / dimensionality reduction was associated with significantly higher values for specificity (*p* = 0.048) and accuracy (0.043).

**Table 4:** Association of study characteristics with four selected performance metrics. Mean values of each metric are provided for different groups for selected study characteristics, along with *p*-values indicating the significance of the associations. Statistically significant *p*-values are highlighted in bold and with an asterisk. For the data processing methods the group 0 indicates that the given data processing method was not applied and group 1 indicates that it was applied. Note: not all studies reported all four performance metrics. As a result, mean values for sensitivity, specificity, accuracy, and AUC were calculated from different subsets of studies

Study characteristic	Sensitivity	Specificity	Accuracy	AUC
Condition (AST, CAN, CO, DEM, DI, HD, HT, PD, RA)	0.83, 0.97, 0.79, -, 0.77, 0.47, 0.78, 0.84, 0.78 (*p* = 0.334)	0.80, 0.99, 0.89, 0.97, 0.85, 0.96, 0.89, 0.89, 0.89 (*p* = 0.951)	0.87, 0.98, 0.81, 0.93, 0.81, 0.93, -, 0.89, 0.83 (*p* = 0.494)	0.85, 1.00, 0.76, 0.96, 0.80, 0.88, 0.78, 0.89, - (*p* = 0.092)
Algorithm (DNN, LG, SVM, TREE, Other)	0.83, 0.76, 0.82, 0.74, 0.80 (*p* = 0.808)	0.95, 0.91, 0.81, 0.75, 0.89 (*p* = 0.176)	0.90, 0.84, 0.85, 0.83, 0.86 (*p* = 0.690)	0.85, 0.81, 0.82, 0.77, 0.83 (*p* = 0.649)
Dataset size (10, 100, 100+, 50)	0.77, 0.78, 0.79, 0.85 (*p* = 0.710)	0.83, 0.87, 0.85, 0.92 (*p* = 0.852)	0.91, 0.85, 0.87, 0.87 (*p* = 0.913)	0.91, 0.82, 0.84, 0.80 (*p* = 0.476)
Validation (EX, LOOCV, Simple, k-fold, Other)	0.82, 0.83, 0.76, 0.75, 0.78 (*p* = 0.817)	0.89, 0.80, 0.91, -, 0.82 (*p* = 0.452)	0.83, 0.92, 0.87, -, - (*p* = 0.167)	0.84, 0.82, 0.85, 0.72, 0.79 (*p* = 0.309)
Labeling (E, EL, F, MD, PM, PRO, VT)	0.71, 0.89, 0.76, 0.94, 0.82, 0.90, 0.85 (*p* = 0.121)	0.79, 0.92, 0.93, 0.97, 0.86, 0.92, 0.93 (*p* = 0.518)	0.81, 0.92, 0.89, 0.89, 0.88, 0.86, 0.88 (*p* = 0.750)	0.78, 0.90, 0.84, 0.89, 0.81, 0.79, 0.90 (*p* = 0.381)
Outcomes (DD, DE, DS, Other)	0.78, 0.80, 0.80, 0.83 (*p* = 0.891)	0.86, 0.93, 0.81, 0.87 (*p* = 0.800)	0.87, 0.85, 0.81, 0.86 (*p* = 0.952)	0.82, 0.86, 0.80, 0.77 (*p* = 0.358)
Year (2014–2017, 2018–2021, 2022–2024)	0.78, 0.78, 0.85 (*p* = 0.500)	0.84, 0.87, 0.89 (*p* = 0.792)	0.83, 0.86, 0.89 (*p* = 0.513)	0.81, 0.78, 0.86 (*p* = 0.080)
Feature engineering (0, 1)	0.87, 0.76 (*p*=0.031*)	0.85, 0.88 (*p* = 0.718)	0.97, 0.84 (p$<$0.001*)	0.84, 0.81 (*p* = 0.508)
Signal denoising (0, 1)	0.81, 0.77 (*p* = 0.511)	0.86, 0.89 (*p* = 0.658)	0.88, 0.83 (*p* = 0.382)	0.83, 0.78 (*p* = 0.246)
Downsampling (0, 1)	0.80, 0.83 (*p* = 0.555)	0.87, 0.85 (*p* = 0.690)	0.86, 0.88 (*p* = 0.439)	0.81, 0.95 (*p*=**0.010** *)
Feature selection (0, 1)	0.78, 0.88 (*p* = 0.067)	0.85, 0.94 (*p*=**0.029** *)	0.85, 0.91 (*p*=**0.048** *)	0.80, 0.88 (*p*=0.043*)

## Discussion

This review summarized 76 studies that applied ML to the monitoring of chronic diseases, offering insights into trends and limitations. Parkinson’s disease (PD) was the most frequently studied condition, likely due to the feasibility of tracking motor symptoms like freezing of gait through wearable sensors. For instance, simple devices capturing accelerometer data were sufficient to detect PD-related motor fluctuations [91]. This contrasts with the absence of studies on liver or kidney disease, which may require more invasive or expensive monitoring methods, making them less suited for remote monitoring.

Wearable devices were most often used as sensors with raw accelerometer data as the most prevalent input parameter type, which is consistent with the high number of PD-related studies. While raw accelerometer data were the most often used parameter overall, heart rate was the most broadly applied parameter as it was monitored in eight different conditions. Heart rate cannot only indicate abnormalities of the cardiovascular system but it may also indicate an increase in sympathetic activity and a decrease in parasympathetic activity which is known to happen in a variety of different chronic conditions [92]. For instance, heart rate variability has been shown to be a crucial marker in the management of chronic diseases such as diabetes [93], hypertension [94], rheumatoid arthritis [95], and COPD [96].

The most frequent outcome were disease exacerbations. The focus on predicting disease exacerbations over diagnosis may stem from the fact that exacerbations offer more frequent data points for ML models to process. Exacerbations provide multiple data endpoints per patient, which contrasts with disease diagnosis data that consist only of a single data point. Patient-reported outcomes (PROs) were the most common labeling method, providing an accessible yet imperfect data source. PROs are comparatively easy to obtain and may contribute to an improved quality of care by focusing on the patient’s perspective. However, one also has to be aware of the biases and drawbacks associated with PROs such as the social desirability bias [97] or the problem that lengthy questionnaires may decrease patient adherence or may even not be feasible at all for some patient groups [98]. Furthermore, Crowe et al. [85] directly compared the accuracy of their prediction algorithms for detecting PD motor symptoms on both expert-labelled data and PROs and showed that the performance with PRO labels was 12–20% lower.

Finally, the analysis on the relation of study characteristics with prediction performance yielded some interesting and quite unintuitive results. We showed that studies that did not perform any feature engineering were associated with a significantly higher accuracy. We believe that methodological shortcomings of some studies not applying feature engineering may be the cause and we provide a short analysis in the Supplementary data. We observed that 19 out of 76 studies did not report any form of cross-validation or alternative validation strategy, which may increase the risk of overfitting and performance overestimation [99]. Finally feature selection was significantly associated with a better performance for three of the four classification metrics. Indeed, prior studies have shown that reducing the dimensionality of the dataset leads to improved generalizability of the model and ultimately improves its predictive power [100].

Our systematic review revealed both promising developments and significant methodological challenges in the application of machine learning for chronic disease monitoring. The PROBAST risk of bias assessment highlighted substantial quality concerns, with 73.3% of studies classified as high risk, primarily due to limitations in the analysis domain. It is important to note that a designation of ‘high risk of bias’ indicates a potential for bias due to methodological shortcomings, rather than definitive evidence that a study is actually biased. These findings align with those of a prior study [101] that found that 90% of studies using ML for perioperative medicine showed either high or unclear risk of bias. The high risk of bias in the analysis domain of PROBAST further emphasizes the need for more robust validation practices, although we acknowledge that some degree of risk of bias is often unavoidable in real-world clinical research, stemming from factors such as limited patient sample sizes or patient withdrawal. The notable variability in methodologies, outcome reporting, and performance metrics across studies presents a significant barrier to generalizing findings and contributes to the reproducibility crisis in healthcare ML applications [102]. We thus advocate for stricter adherence to established reporting guidelines, particularly the TRIPOD-AI (Transparent Reporting of a multivariable prediction model for Individual Prognosis Or Diagnosis - Artificial Intelligence) framework, which provides comprehensive standards for ML applications in healthcare [103]. These guidelines, together with thorough risk of bias assessments using PROBAST, form a robust foundation for high-quality ML research. Additionally, sharing programming code and anonymized patient data when feasible would significantly enhance trust and reproducibility in this field.

### Future directions and ethical considerations

The demand for remote patient monitoring (RPM) systems has increased significantly in recent years, driven by demographic shifts and rising workloads for healthcare professionals. The growing use of wearable devices, coupled with advancements in artificial intelligence, enhances the potential of RPM systems for continuous patient care and early diagnosis. For example, in a recent study on dementia management, RPM systems helped detect abnormal vital signs, ranging from fever indicative of infection to bradycardia caused by medication, offering a proactive approach to patient care [104].

The aim of RPM has to be to be integrated into Clinical Decision Support Systems (CDSS), which will assist healthcare providers in making data-driven clinical decisions for diseased patients. Continuous real-time data from wearables can feed into CDSS, enhancing their capacity to provide timely and accurate medical recommendations. However, to ensure clinical relevance, future research should focus not only on improving the accuracy of disease predictions but also on making these predictions interpretable and actionable for healthcare providers. This is particularly important for CDSS, which rely on visualizing decisions or validating them through domain expertise [11].

Alongside the technical advancements, the ethical implications of ML and remote monitoring in healthcare must be thoroughly considered. As ML systems increasingly influence patient care, it is crucial that these systems remain transparent, understandable, and trustworthy for both clinicians and patients. Ensuring patient consent and protecting patient autonomy are key, especially as RPM and CDSS become more prevalent. The process of obtaining data from personal devices or healthcare providers can be complex and time-consuming, raising concerns about accessibility and transparency [105].

Additionally, the opacity of “black-box” ML algorithms can erode trust in medical decisions made by such systems, underscoring the need for explainable AI. Data security is another critical concern; robust measures must be in place to prevent unauthorized access and ensure the privacy of sensitive health data [106]. Moreover, ML models can perpetuate existing healthcare disparities if not properly managed, particularly when biased training data is used [107]. Ethical guidelines should be developed to address these challenges, emphasizing patient consent, data privacy, and equitable access to healthcare innovations, ensuring that CDSS systems are deployed with fairness and responsibility.

### Limitations

In this systematic review there were several limitations. The search string was long and included a predefined selection of chronic conditions excluding some notable chronic diseases such as autoimmune disease or Huntington disease. This was due to the great amount of studies found initially, which prompted us to narrow our scope to focus on chronic diseases mostly influenced by lifestyle and the environment. 14 of the 76 studies were manually added, and it cannot be ruled out that during reading through the related works sections and other related reviews, some eligible studies were overlooked. Finally, in any literature-based study, human error during study selection, tagging or risk of bias assessment is inevitable, and differing interpretations of articles can lead to disagreements between raters. Many PROBAST questions are subjective, making consistent judgment even more challenging. Large Language Models (LLMs) may offer a promising solution to reduce human error. In the Supplementary data we present our experiments for LLM-guided study selection, study tagging and risk of bias assessment 6.

## Conclusion

This systematic review of 76 studies on ML applications for remote monitoring of chronic disease revealed significant methodological concerns, with PROBAST assessment showing overall high risk of bias in 73.7% of studies. Despite these quality limitations, we identified clear patterns: Parkinson’s disease, COPD, and diabetes were the most studied conditions, primarily using wearable devices for movement and heart rate data, with tree-based models as the predominant analytical approach. Several chronic conditions, including liver disease and autoimmune disorders, remain unexplored in this context. The high proportion of studies with methodological limitations emphasizes the need for standardized approaches following established guidelines such as TRIPOD-AI. Nevertheless, the evidence suggests that remote monitoring combined with ML holds substantial promise for chronic disease management. When implemented with robust methodology, this approach has the potential to enhance patient care, reduce complications, and advance our understanding of chronic conditions.

## Figures and Tables

**Fig. S1:**
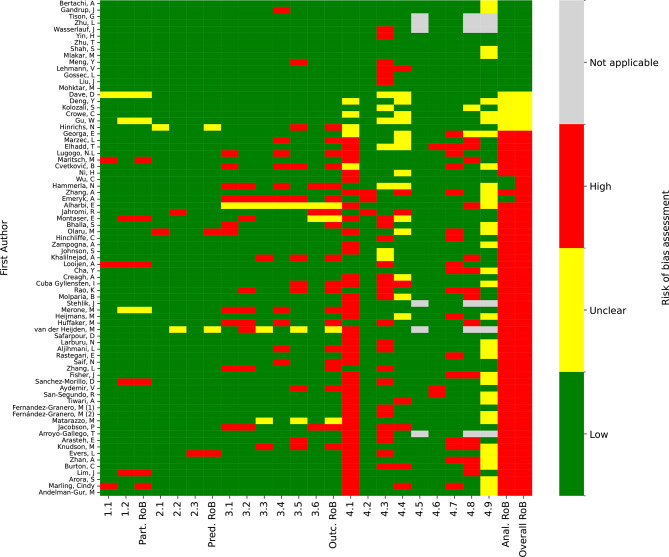
Risk of bias assessment for every individual study

## Data Availability

The study tagging file, the risk of bias assessment file and the Python script for the analyses can be found on this Github repository: https://github.com/Prgrmmrjns/Systematic-review-RPM-ML.

## Supplementary data

### Risk of bias assessment

#### Confounders between feature engineering and accuracy

We performed an additional analysis on potential confounders that may explain why the accuracy of studies that did not perform any feature engineering was significantly better (average accuracy without feature engineering: 0.97, with feature engineering: 0.84, *p* < 0.001) than the accuracy of studies that performed feature engineering. There were only five studies that did not perform feature engineering and reported an accuracy score while there were 26 studies performing feature engineering and reporting an accuracy score. All of these five studies displayed an overall high risk of bias. The studies all had a comparatively small dataset size with one study [67] only having one study subject. In addition, three of the five studies only performed a simple validation split. We thus believe that the significantly higher reported accuracy for studies not performing feature engineering can be at least partially explained by methodological shortcomings.

### Experimenting with a large Language model for study selection, tagging and risk of bias assessment

Deciding whether studies meet all inclusion criteria, correctly tagging study characteristics and assessing the risk of bias are all arduous tasks requiring expert knowledge and often take a long time. The advent of Large Language Models (LLMs) has opened up the possibility for accelerating and automating these tasks. LLMs combined with retrieval augmented generation (RAG), with their ability to process and analyze vast amounts of text, can potentially reduce the time and effort required for systematic review tasks that traditionally rely heavily on expert input. In three experiments, we assessed the capability of NotebookLM which is based on the Gemini 1.5 model by Google to perform study selection, study tagging and risk of bias assessment and compare the results with our own decisions.

For study selection we randomly selected five studies that we included as well as 5 randomly selected studies from our literature search that we considered for full-text screening but eventually did not include. We gave the eligibility criteria mentioned in Table 2 as well as the question whether the study should be included or not according to the eligibility criteria. NotebookLM correctly assessed the study selection criteria in 6 out of 10 presented studies. Of the five studies that were not selected by us NotebookLM falsely took the decision to include three of these. For example, we did not include the study by Honkoop et al. [108] as the study does not report any Machine learning results. Yet, NotebookLM explained itself as follows regarding this aspect: “ML applied and ML methodology sufficiently described: While the specific ML algorithms are not detailed in the excerpt, the study mentions using techniques like cluster, spectral, and factor analyses, along with probabilistic methods and data mining. It also highlights the involvement of collaborators experienced in handling continuous data for predictive modeling. This suggests a clear application of ML with sufficient methodological description.” As included study we randomly selected the study SugarMate [44]. NotebookLM falsely assessed that this study should not be included as it did not report relevant outcomes. Yet, the study uses their prediction model to classify current glucose levels into four categories including hypo- and hyperglycemic ranges.

When it comes to study tagging NotebookLM was mostly able to reproduce the study tags that we generated for the randomly selected study SugarMate [44]. We assessed hypoglycemia and hyperglycemias as a disease exacerbation as we defined it as acute and temporary disease state. Yet NotebookLM suggested that there were in fact three outcomes in the study: disease severity, disease risk and disease exacerbations. Similarly, for tagging the ground truth labeling method in the article NotebookLM suggested that there were four different labeling methods used while in fact only parallel measurement using a CGM device was used.

NotebookLM was also used to create a PROBAST risk of bias assessment for the Sugarmate study. NotebookLM assessed the study to be of overall unclear risk, exactly how we assessed the study. NotebookLM responded to two signaling questions with Unclear. NotebookLM both assessed signaling questions 1.2 and 4.3, with Unclear as there were no mentions of eligibility criteria and excluded patients, respectively.

In summary, while LLMs like NotebookLM can provide valuable support in systematic review tasks by summarizing text or suggesting tags, their performance is rather unsatisfying, particularly for study selection (Accuracy = 0.6). This is in line with another study [109] showing that GPT4 had an accuracy of 0.54 for the full-text screening process. Yet, we believe that many of the problems leading to misconceptions by the LLM could be avoided by providing more structured prompts including author-guided instructions or rules, tailored specifically to the systematic review at hand.

## Abbreviations

AECOPD: Acute Exacerbation of Chronic Obstructive Pulmonary Disease
AI: Artificial Intelligence
ANOVA: Analysis of Variance
AUC: Area Under the Receiver Operating Characteristic Curve
BN: Bayesian Networks
CART: Classification and Regression Tree
CCA: Canonical-Correlation Analysis
CDSS: Clinical Decision Support System
CGM: Continuous Glucose Monitoring
CNN: Convolutional Neural Network
COPD: Chronic Obstructive Pulmonary Disease
DNN: Deep Neural Network
DE: Disease Exacerbations
DD: Disease Detection
DH: Disease Hospitalisation
DM: Disease Mortality
DS: Disease Severity
DT: Decision Tree
EM: Ensemble Model
GB: Gradient Boosting Tree
HM: Hybrid Model
HMM: Hidden Markov Model
HUM: Humidity
IF: Isolation Forest
KAF: Kernel Adaptive Filters
KNN: K-Nearest Neighbors
KM: K-Means Clustering
LG: Logistic / Linear Regression
LOOCV: Leave-One-Out Cross-Validation
MACD: Moving Average Convergence/Divergence
ML: Machine Learning
MM: Mixed Model
NB: Naive Bayes
PD: Parkinson’s Disease
PLCA: Probabilistic Latent Component Analysis
PN: Petri Net
PNN: Probabilistic Neural Network
PRISMA: Preferred Reporting Items for Systematic Reviews and Meta-Analyses
PRO: Patient-Reported Outcome
PROBAST: Prediction Model Risk of Bias Assessment Tool
RB: Rank-Based Machine Learning
RF: Random Forest
RNN: Recurrent Neural Network
RoB: Risk of Bias
RPM: Remote Patient Monitoring
SD: Subspace Discriminant Classifier
SBM: Similarity-Based Modeling
SEN: Sensitivity
SPE: Specificity
SVM: Support Vector Machine
TRIPOD-AI: Transparent Reporting of a Multivariable Prediction Model for Individual Prognosis or Diagnosis - Artificial Intelligence
WB: Patient Wellbeing
